# Systematic Review on Outbreaks of SARS-CoV-2 on Cruise, Navy and Cargo Ships

**DOI:** 10.3390/ijerph18105195

**Published:** 2021-05-13

**Authors:** Ann-Christin Kordsmeyer, Natascha Mojtahedzadeh, Jan Heidrich, Kristina Militzer, Thomas von Münster, Lukas Belz, Hans-Joachim Jensen, Sinan Bakir, Esther Henning, Julian Heuser, Angelina Klein, Nadine Sproessel, Axel Ekkernkamp, Lena Ehlers, Jens de Boer, Scarlett Kleine-Kampmann, Martin Dirksen-Fischer, Anita Plenge-Bönig, Volker Harth, Marcus Oldenburg

**Affiliations:** 1 Institute for Occupational and Maritime Medicine (ZfAM), University Medical Center Hamburg-Eppendorf (UKE), 20459 Hamburg, Germany; n.mojtahedzadeh@uke.de (N.M.); jan.heidrich@justiz.hamburg.de (J.H.); kristina.militzer@justiz.hamburg.de (K.M.); thomas.vonmuenster@justiz.hamburg.de (T.v.M.); lukas.belz@justiz.hamburg.de (L.B.); hans_joachim-jensen@t-online.de (H.-J.J.); harth@uke.de (V.H.); marcus.oldenburg@justiz.hamburg.de (M.O.); 2 Department of Trauma and Reconstructive Surgery and Rehabilitative Medicine, Medical University Greifswald, 17475 Greifswald, Germany; sinan.bakir@uni-greifswald.de (S.B.); Esther.Henning@med.uni-greifswald.de (E.H.); Julian.Heuser@med.uni-greifswald.de (J.H.); ak166297@uni-greifswald.de (A.K.); Nadine.Sproessel@med.uni-greifswald.de (N.S.); traumato@uni-greifswald.de (A.E.); 3 Hamburg Port Health Center (HPHC), Institute for Hygiene and Environment, 20537 Hamburg, Germany; lena.ehlers@hu.hamburg.de (L.E.); jens.deboer@hu.hamburg.de (J.d.B.); scarlett.kleine-kampmann@hu.hamburg.de (S.K.-K.); martin.dirksen-fischer@hu.hamburg.de (M.D.-F.); 4 Infectious Diseases Surveillance Center, Institute for Hygiene and Environment, 20539 Hamburg, Germany; anita.plenge-boenig@hu.hamburg.de

**Keywords:** cargo ships, COVID-19, cruise ships, navy vessels, outbreak management, SARS-CoV-2

## Abstract

The confined environment of a ship promotes the transmission of the severe acute respiratory syndrome coronavirus type 2 (SARS-CoV-2) due to close contact among the population on board. The study aims to provide an overview of outbreaks of SARS-CoV-2 on board of cruise, navy or cargo ships, to identify relevant outbreak management techniques, related problems and to derive recommendations for prevention. Four databases were searched. The study selection included reports about seroprevalences or clinically/laboratory confirmed infections of SARS-CoV-2 on board ships between the first of January, 2020 and the end of July, 2020. A total of 37 studies were included of whom 33 reported outbreaks of SARS-CoV-2 on cruise ships (27 studies referred to the Diamond Princess). Two studies considered outbreaks on the Grand Princess, three studies informed about Nile River cruises and one study about the MS Westerdam (mention of multiple outbreaks possible in one study). Additionally, three studies reported outbreaks of SARS-CoV-2 on navy vessels and one study referred to a cargo ship. Problems in handling outbreaks resulted from a high number of asymptomatic infections, transportation issues, challenges in communication or limited access to health care. Responsible operators need to implement infection control measures which should be described in outbreak management plans for ships to prevent transmission risks, including, e.g., education, testing strategies, communication lines, social distancing and hygiene regulations.

## 1. Introduction

Globalization results in various new challenges regarding the epidemiology of infectious diseases. Developing new markets constantly leads to a growing commerce of goods and services. Moreover, increased mobility results in a faster and more extensive spread of infectious diseases [1]. Growing maritime traffic and voyages play an important role in this context. In 2019, about 29.7 millions of passengers on cruise ships were registered internationally [2] from whom more than 7.7 million were European cruise ship passengers [3]. In distinction from that, in 2009, only 17.8 million international passengers were recorded on cruise ships [4]. In a confined ship environment with different types of social events e.g., when sharing a dinner table there is forcibly a narrow contact between passengers and crew members with different travel histories for a longer period of time. Consequently, different types of ships can depict a fitting milieu for the transmission of infections between persons. They can be the source of an outbreak of a disease as well as a promoter of transmission of already introduced pathogens on board. The spread of vectors and microbes from ships to land-based populations can also be encouraged [5]. Furthermore, crew members and passengers normally come from different countries which may have varying risks and occurrence of infectious diseases as well as different types of vaccination strategies [6]. In addition to the growing cruise industry, container ship traffic is also increasing. In 2019, about 152 millions of TEU (twenty foot equivalent unit, standard container size) were transported internationally [7], representing the highest growth since 2014 [8]. According to prognoses there will be about 175 TEU for 2023 [7].

In the past, several outbreaks of infectious diseases were reported on ships particularly referring to norovirus-associated gastrointestinal diseases e.g., [9,10,11,12,13,14] or respiratory diseases like Influenza A or B, e.g., [15,16,17,18,19]. There are also scientific publications available regarding vaccine-preventable viral diseases, such as measles, chickenpox or rubella [20,21,22]. Regarding, for instance meningococcal infections disease in the maritime sector, there hardly exist any published scientific reports so far [23,24].

In December 2019, a new type of coronavirus (severe acute respiratory syndrome coronavirus type 2, SARS-CoV-2) was discovered for the first time in Wuhan, China. Ever since, the virus has been spreading rapidly on a global scale [25,26]. SARS-CoV-2 has been identified as the causative agent of coronavirus disease 2019 (COVID-19) and arises via respiratory intake of virus-containing particles (e.g., during breathing, coughing, talking, singing, and sneezing). The most commonly recorded symptoms include cough, fever, rhinitis, and loss of smell and taste [27]. In an international review, the proportion of deceased patients among those treated with intensive care was estimated to be 34% [28]. Further progression of the pandemic resulted due to the appearance of new variants such as B.1.1.7 (spread from Great Britain since September 2020) which is more easily transmissible and associated with higher age-independent mortality, B.1.351 (reported from South Africa in December 2020), which is recognized as less effective against available vaccination or the P.1 variant (examined from the Brazilian state Amazonas in January 2021), which also appears to be more contagious and also less effective against introduced vaccines [29,30]. Whether and to what extent previously approved vaccines can protect against new variants cannot be predicted [31].

First infections of SARS-CoV-2 on cruise ships were already noted in the beginning of February 2020, e.g., [32]. Since governments and ports prevented various cruise ships from docking or recommended to refrain from cruise voyages, cruise lines mostly suspended their operations (in contrast to cargo ships). As a further example, cruise operations to and from ports of the United States of America (U.S.A.) were even prohibited since mid-March 2020. In November 2020, a replaced order covered by a new “conditional sailing order” has been put in place, which allows cruise lines to offer voyages in U.S. waters under consideration of a comprehensive set of regulations. Before restarting cruise operations, responsible companies are obligated to conduct a series of simulated cruises without passengers including drills for on board infection incidents [33].

Within the framework of the present paper the current state of research regarding SARS-CoV-2 outbreaks on ships sailing worldwide, its management techniques and related problems were evaluated.

## 2. Materials and Methods

### 2.1. Research Question and Objectives

The aim of this systematic literature review was threefold: (I) to provide an overview of observed SARS-CoV-2 outbreaks on ships sailing worldwide, (II) to identify relevant outbreak management techniques and related problems as well as (III) to derive recommendations for actions regarding on board prevention and control approaches.

### 2.2. Search Strategy and Identification of Relevant Studies

To detect relevant studies, four electronic databases were included using PubMed, MEDLINE, Web of Science, and CINAHL. The databases were searched for the first seven months of 2020 (until 31 July 2020). The search strategy included two main topics referring on the one hand to COVID-19 as a public health event and on the other hand to the maritime setting guided by the handbook for management of public health events on board ships [34], using the subsequent terms: (COVID-19, searched as a supplementary concept including, for instance entry terms like 2019-nCoV infection or SARS-CoV-2 infection) AND (ship OR cargo ship OR vessel OR boat OR navy ship OR fishing vessel OR cruise OR passenger OR yacht OR merchant ship OR ferry OR harbor OR port). The search strategy was developed for PubMed and afterwards adapted for the other databases. In addition, a hand search was performed considering databases of the European Centre for Disease Prevention and Control (ECDC), the United States Centers for Disease Control and Prevention (US-CDC) and Google Scholar. Finally, reference lists of the included studies were searched.

### 2.3. Study Selection

To achieve the research goal, it was necessary to define certain inclusion and exclusion criteria according to the PEO-criteria by Khan et al. [35] (Population, Exposition and Outcome, displayed in Table 1). A systematic review by Mouchtouri et al. [36] served as a further orientation for the definition of the inclusion and exclusion criteria for the maritime setting.

Referring to the study design, cross-sectional or single case studies were included, when being published in journal articles, reports and national or international publications. Duplicates and comments without own data were excluded. Only studies in the languages English or German were searched. Titles and abstracts were screened and afterwards full texts were assessed for eligibility by two reviewers independently (A.-C.K. and N.M.). Information on the inter-rater reliability was given by means of Cohen’s kappa. 

### 2.4. Data Extraction and Analysis

Included studies and reports were charted using data extraction sheets including the author, title, country of port called, study design, methodology, setting, vessel type, sample size, population, main measurements, aims of the study and main results. Data were collected by two reviewers independently (A.-C.K. and N.M.). Studies describing the same outbreak on were summarized in a narrative way including descriptive and numerical data of outbreaks of SARS-CoV-2 on board ships, approaches for outbreak management, and occurred problems. Results were oriented towards the PRISMA guideline (preferred reporting items for systematic reviews and meta-analyses) for systematic reviews maintaining essential reporting items [37]. A study protocol was not prepared.

## 3. Results

**Figure 1:**
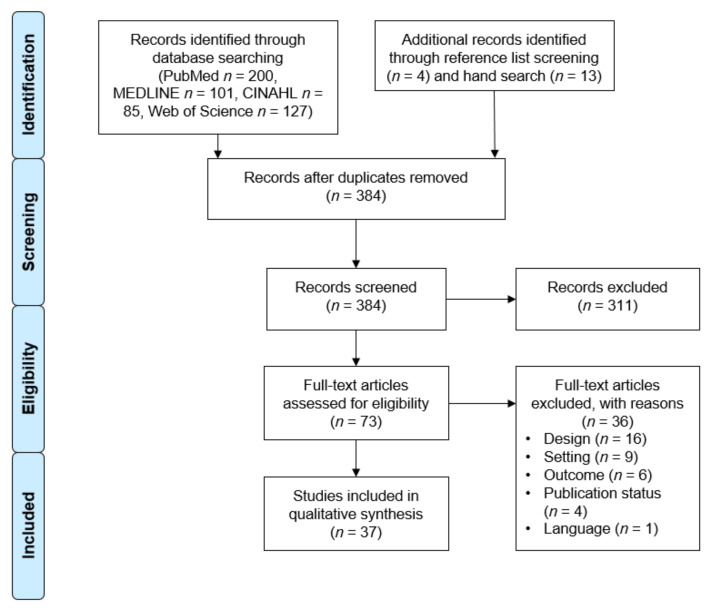
PRISMA Flow Diagram for the selection process of systematic reviews, following Moher et al. [37].

All in all, 513 studies were identified within the four databases searched, followed by 17 additional records through hand search and the screening of reference lists. After duplicate exclusion and a title and abstract screening, 73 studies were analyzed for eligibility. Studies were excluded according to the previously defined criteria (i.e., no reference to maritime context or no confirmed infections with SARS-CoV-2). A moderate Cohens-Kappa of 0.78 shows a substantial agreement between the two reviewers (A.-C.K. and N.M.) based on Landis and Koch [38]. To sum up, Figure 1 presents the process of study selection.

### 3.1. Study Characteristcs

As shown in Table 2, 27 studies reported called ports in Japan, three studies referred to called ports in Egypt (Nile river cruises) and five studies informed about ports of call in the U.S.A. Two studies were included from Australia without naming a reference to a specific ship or called port. One study was identified from a port of call in Cambodia and China each. Most of the included studies (*n* = 33) reported outbreaks of SARS-CoV-2 on cruise ships. Overall, up to two ships with outbreaks of SARS-CoV-2 could be mentioned in one study. Supplementary Material concerning background information on the included studies can be accessed from Table S1.

### 3.2. COVID-19 on Cruise Ships

#### 3.2.1. Diamond Princess

Overall, 27 studies were identified referring to the Diamond Princess. Eleven studies gave a descriptive epidemiology report of the outbreak of SARS-CoV-2 on the cruise ship [39,40,41,42,43,44,45,46,47,48,49] analyzing, e.g., the proportion of asymptomatic cases [39,42,43,44,45,46,47,48,49]. Seven studies analyzed clinical outcomes or clinical courses [39,45,50,51,52,53,54] e.g., with a special focus on conjunctivitis [53], lung opacities and airway abnormalities [39,52] or single patients [51]. Two studies each used analysis of genome sequences [44,55] or SARS-CoV-2 seroconversion [56,57], seven studies used mathematical modelling of infectious diseases [32,58,59,60,61,62,63] and one study reported on medical transport of passengers [64].

##### Course of the Outbreak

**Figure 2:**
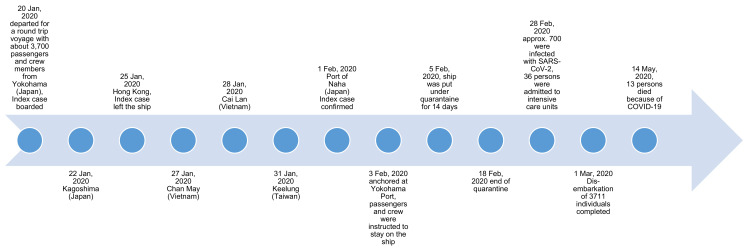
Course of the SARS-CoV-2 outbreak on the Diamond Princess.

Several authors [32,39,40,41,42,43,44,45,46,47,48,49,51,52,53,54,55,56,57,58,59,60,61,62,63,64,65] described that the Diamond Princess departed from Yokohama on 20 January 2020 for a 16-day journey including destinations in Japan, Hong Kong, Vietnam, and Taiwan, accommodating about 3700 passengers and crew members (Figure 2). However, it was reported that an 80-year-old passenger who disembarked in Hong Kong on 25 January 2020 was presented with mild dry cough two days earlier (23 January 2020) [32,40,41,42,43,44,46,47,48,49,52,55,56,58,60,61,62,63,64,65]. Confirmed positive test results for SARS-CoV-2 for this index case were available on 1 February followed by the first 10 people diagnosed with a SARS-CoV-2 infection in the subsequent two to four days depending on the reference [32,40,41,43,44,46,47,48,49,51,54,55,56,58,61,62,63,64]. Consequently, Japanese authorities put the Diamond Princess under quarantine for 14 days restraining passengers to their cabins whereby the passenger cabin capacity ranged from one to four persons [32,40,41,42,43,44,46,47,48,49,51,52,54,56,58,60,61,62,63,64,65]. Passengers were authorized to spend time on an external deck each day for 60 minutes on the condition to wear masks, keep the distance and not touch any surfaces. Hence, regular duties like food and cleaning services needed to be maintained during the quarantine by crew members, who were restricted to remain in their cabins when having COVID-19 specific symptoms [32,41,42,44,46,47,48,49,56,58,65]. Additionally, on 7 February, thermometers were distributed asking the passengers and crew members to monitor their own temperature multiple times a day calling a “Fever Call Centre” in case of abnormalities. After informing the hotline, members of the Japan’s disaster medical assistance team (DMAT) and Ministry of Defence visited passengers for an examination of their health condition and if necessary, oropharyngeal swabs were taken from suspected cases [46,48,49,50].

Initially, all passengers and crew members with noticeable symptoms of COVID-19 like fever, cough or sore throat or contact persons to those with a confirmed infection were tested for SARS-CoV-2 by using polymerase chain reaction (PCR) assays [41,42,43,44,48,49,55,56,58,64]. Due to limited test capacities, testing was expanded only after 11 February 2020 from a symptom-based testing approach of those with COVID-19-related symptoms to an approach starting with older or multi-morbid individuals as well as those accommodated in internal cabins. Persons on board who were tested positive were transferred and isolated in local hospitals whereby those with a negative test result remained on board restricted to their cabins [32,42,43,44,45,49,52,56,57,58,63,65]. Though, this approach did not include crew members, who had to complete a 14-day quarantine on the cruise ship, were repatriated and followed home country specific-instructions or completed a 14-day quarantine ashore in Japan after disembarkation of all passengers [42,43]. Home nations were allowed to repatriate their passengers after 17 February 2020 including many countries like the U.S., Canada, Hong Kong, Australia, and Korea [32,42,43,44,51,56]. Overall, 712 persons had been infected with SARS-CoV-2 (reported until 17 March 2020) on the Diamond Princess, including 36 persons who were admitted to intensive care units (until 28 February 2020), and 13 persons who died (until 14 May 2020) because of COVID-19 (Figure 2) [32,40,41,43,44,45,48,52,54,55,56,57,58,59,60,63,64,65]. 

##### Analysis of Asymptomatic and Symptomatic Cases

Nine studies reported about the proportion of asymptomatic individuals of the Diamond Princess [39,44,45,46,47,48,49] ranging between 14% (Yamagishi et al. [46], of a total of 172 confirmed SARS-CoV-2 infections) and 73% (Inui et al. [39], of a total of 104 confirmed SARS-CoV-2 infections), whereby studies with a sample size of 500 individuals and more reported between 46.5% and 58.9% of asymptomatic courses [42,47,48,49]. Overall, it was reported that high rates of confirmed asymptomatic cases were traced back to the systematic testing of passengers starting on 14 February 2020 [49].

Clinical outcomes or clinical courses of symptomatic cases were mostly presented in case studies, as applied to the probable index case of the Diamond Princess in a study by Leung et al. [51]. The authors summarized that although the case was presented as the first passenger with a confirmed diagnosis, the origin of the outbreak remains unclear. It could be possible, that the described patient acquired the infection during the stay on board, since a transmission from asymptomatic cases has been reported [51]. Moreover, two crew members were examined as examples of the course of COVID-19 in young and healthy persons. Arashiro et al. [65] presented one restaurant server and one kitchen cleaner with mild upper respiratory symptoms without identifiable pneumonia [65]. 

To shed more light on pneumonia, a study conducted by Kato et al. [54] contrasted 70 cases with and without pneumonia. The clinical parameters elevated body temperature, heart rate, and respiratory rate, increased lactate dehydrogenase (LDH), aspartate aminotransferase (AST), and C-reactive protein (CRP) levels as well as lower serum albumin level and lymphocyte count were linked with a pneumonia. Almost every patient with a pneumonia in the study (97.7%) presented with ground-glass opacities (GGO) on X-ray. Fourteen patients with a pneumonia were mechanically ventilated for 12 days (median duration) and two patients died [54]. Similar results with respect to LDH were obtained by Tabata et al. [45] describing significantly higher levels in ten cases who developed COVID-19 specific symptoms contrasted to those 33 cases who did not develop any symptoms during the study (*n* = 104; asymptomatic, mild, and severe symptoms in 43, 41, and 20 patients on admission and 33, 43, and 28 patients at the end of observation). For cases with a severe course of COVID-19, older age, consolidation on chest computed tomography (CT), as well as lymphopenia were more prevalent compared to patients with mild symptoms and therefore evaluated as possible risk factors [45]. The latter results could also be replicated in a study by Inui et al. [39] analyzing pulmonary parameters of asymptomatic (73%, *n* = 76) and symptomatic (27%, *n* = 28) cases of a total of 104 participants [39]. 

Further clinical parameters were analyzed in a sample of 17 cases including cough and fever as main symptoms, high blood CRP levels and bilateral GGO mainly located in the peripheral lung in chest CT evaluation. Three cases were classified as critical and one of which died as a result of multiple organ failure [52]. Apart from that, one study presented a case of severe viral conjunctivitis of a passenger disembarked from the Diamond Princess [53].

Phylogenetic analysis was performed in two studies analyzing the outbreak of the Diamond Princess [44,55]. Whole-genome sequencing of SARS-CoV-2 revealed that the spreading of the virus was initiated by a single introduction prior to the implementation of the quarantine measures. Overall, no specifics were observed related to a certain area of the ship since the persons on board were widely disseminated across the ship. However, some subordinate clusters could be associated with recreational mass-gathering events like sharing a dinner table or infections among passengers being accommodated in the same cabin [55]. Additionally, Plucinski et al. [44] presented an attack rate of individuals in single-person cabins of 18% in contrast to 63% and 81% in a shared cabin with an asymptomatic or symptomatic SARS-Cov-2-infected person, respectively. The authors stated that a triage by symptoms without considering the cabin status may be deficient to assess the risk for SARS-CoV-2 infection [44]. 

Considering seroconversion and viral shedding of SARS-CoV-2, Hung et al. [56] evaluated that individuals infected with SARS-CoV-2 disembarking the Diamond Princess without any symptoms might seroconvert having an elevated viral load and continuing viral shedding. The examined passengers who had viral pneumonia were affected by a higher antibody response than those without ground-glass changes. Overall, functional interaction between PCR and serology was suggested for the identification of cases and contacts. Miyamae et al. [57] also analyzed viral shedding in 23 SARS-CoV-2 infected individuals reporting about asymptomatic or mild courses indicating a median duration of 19 days of viral shedding after recognition of viral activities. Moreover, eight cases received a positive PCR result after they were once tested negative. 

##### Mathematical Modelling

The studies applying mathematical modelling on the basic reproductive number presented heterogeneous results. Zhang et al. [62] calculated that the reproduction number (R0) for SARS-CoV-2 was 2.28 (95% CI: 2.06 to 2.52) at the beginning of the outbreak. Despite the implemented quarantine measures, the authors’ calculation assumed that R0 was still elevated. More drastically results were presented by Röcklöv et al. [32] and Mizumoto and Chowell [63]: the former reported that the basic reproduction rate was found to be 4 times higher on board (to 14.8) in comparison to the reproduction rate in Wuhan (3.7) at the early stage of the outbreak. As in the previous study, the introduced quarantine measures were described as mitigating (without any intervention it was calculated that 79% might have been infected). 2307 estimated cases were prevented by means of public health measures lowering the basic reproduction rate to 1.78. However, an early evacuation of all passengers when arriving in Yokohama at 3 February 2020 would have resulted in an estimated total of 76 infections with SARS-CoV-2 only [32]. Mizumoto and Chowell [63] computed a mean reproduction number of approximately 11 in a closed setting like the Diamond Princes. Again, a decreasing mean reproduction number was reported after the introduction of quarantine measures. Less drastic results on the basic reproductive number were presented by Liu et al. [59] following two assumptions: with intensive social contacts at the early stage of the outbreak the basic reproductive number was estimated to be 6.94, implying an infection of all individuals within one month compared to the implemented quarantine measures resulting in a basic reproductive number of 0.2. 

Besides modelling the reproductive number of the SARS-CoV-2 outbreak of the Diamond Princess, Emery et al. [58] calculated the proportion of asymptomatic passengers (74%), who may have contributed considerably to the spread of the disease (69% of asymptomatic individuals were presented as the source of all infections by means of a transmission model including a- and pre-symptomatic states). In total, the model estimated 1304 infected individuals (35% of the primary Diamond Princess population) whereof 53% of infections had not been detected, predominantly asymptomatic. They either turned negative before they were tested, have not yet been tested or they were not yet detectable at that point. As opposed to that, Mizumoto et al. [60] predicted the asymptomatic proportion to be 17.9% (credible interval (CrI): 15.5–20.2%, a Bayesian framework using Hamiltonian Monte Carlo (HMC) algorithm was applied to estimate the probability of being asymptomatic along with the infection time). Further results described that most of the SARS-CoV-2 infections happened before implementing quarantine measures on 5 February, 2020, underlining transmission activities in confined settings. Other than that, Nishiura et al. [61] followed a back calculation method to evaluate the incidence of SARS-CoV-2 infections after the peak time of infections from 2–4 February 2020. After implementing isolation and quarantine measures on 5 February 2020, the cumulative incidence without close contact was described to be decreasing (prediction with and without close contacts 1373 (95% CI: 570, 2176) and 766 (95% CI: 587, 946), observation 102 and 47 cases), underlining the success of movement restrictions from 5 February 2020 onwards.

##### Medical Transport

Identifying hospitals which were able to admit numerous patients at once and handling medical transport were highlighted as the main challenge, wherefore the DMAT was contacted. The DMAT managed to transport 203 patients to hospitals located in Kanagawa and 566 patients to those in 15 other regions by means of a newly developed system to classify and prioritize COVID-19 patients on the cruise ship [64]. Within the system patients were categorized referring to their medical condition and PCR-results: the first category was divided into two parts: firstly, Category I-1 needed the most urgent treatment independent of a PCR result and patients were transported to emergency rooms near the ship. Secondly, Category I-2 consisted of patients who suffered from severe comorbidities requiring time-critical medical treatment (also independent of PCR results). Those patients were transported to a specialized medical facility on infectious diseases. The remaining two categories consisted of those who were vulnerable for a severe disease progression in case of an infection (Category II, transportation to a quarantine facility after being categorized as asymptomatic in combination with a negative PCR result) and those who received a positive PCR result but were presented as asymptomatic or with mild symptoms (Category III, transportation to a specialized medical facility on infectious diseases in a different region) [64].

#### 3.2.2. Grand Princess

Beside the SARS-CoV-2 outbreak on the Diamond Princess, another ship of Princess Cruises was also affected. In mid-February (11–21) 2020, the Grand Princess started a trip from San Francisco to Mexico, including four stays (Voyage A). In addition, for the next trip (Voyage B) some of the crew members (1111) and passengers (68) continued to travel starting again from San Francisco on 21 February 2020 until 7 March 2020. After informing about two patients with COVID-19-related symptoms disembarked from Voyage A on 4 March 2020 (positive confirmed test result for one person), the cruise line started to cancel group events as a consequence on the ongoing Voyage B. In the meantime, 20 other cases and one death were described in connection with the Voyage A. Therefore, a specialized team was transferred on the ship by helicopter on 5 March 2020 to test 45 passengers and crew members with relevant symptoms. Overall, 21 (46.7%) positive test results were detected (2 passengers and 19 crew members) who were afterwards quarantined in their cabins. As a consequence, public dining was abolished and exchanged by room service. After returning to Oakland, California on 8 March 2020, an ashore quarantine was ordered for passengers and crew members for 14 days, supplemented with a SARS-CoV-2 testing or hospitalization in case of comorbidities or COVID-19-related symptoms. A total of 78 (16.6%) of 469 individuals were identified as positive on 21 March 2020. While repatriation flights were initiated by some governments, other foreigners concluded a ship-based quarantine under the management of the company and public health experts. Afterwards, the ship was disinfected based on the regulations from US-CDC’s Vessel Sanitation Program (VSP) [42].

Additionally, Jorden et al. [66] reported that a transmission of the SARS-CoV-2 virus took place in California traced back to one unidentified person on board of the described cruise ship (either passenger or crew member) which departed in San Francisco on 11 February, 2020 among others [66].

#### 3.2.3. MS Westerdam

Because of a suspected case of COVID-19 with symptoms of pneumonia, the Japanese government prohibited the MS Westerdam two days before arriving in Okinawa on 6 February 2020 from docking at Naha Port because of national safety reasons after travelling from Hong Kong (1 February 2020) through Kaohsiung (5 February 2020) to Ishigaki Port [43]. Under the given circumstances, the cruise ship did not know which port to call at. However, Cambodia authorized the MS Westerdam to dock and disembark the passengers on 12 February 2020. Three days later, a case of COVID-19 was discovered in an American woman (83 years old) as announced by the Malaysian government [43].

#### 3.2.4. Nile River Cruises

Three included studies refer to Nile River cruises [50,67,68]. Overall, Schuchat [68] summarized that 101 persons from nine separate Nile River cruises in the time period between 11 February and 5 March 2020 returned to 18 different states in the United States and were tested positive for SARS-CoV-2. Sekizuka et al. [67] stressed that 26 cases of COVID-19 in Japan were connected to cruise travels on the Nile River in the time span from 5 to 15 March 2020 most of them boarding between Cairo and Luxor for three to four days. Fever and sore throat were noticed when returning to Japan afterwards. Initially, the Egyptian Health authorities informed about 12 COVID-19 cases among Egyptian crew members on a Nile River cruise ship on 6 March. One day later, it was clarified that 45 individuals on board were tested positive for SARS-CoV-2, wherefore a quarantine at a dock in Luxor was implemented. Further genome analysis [67] revealed two possible travel-related clusters in Japan from imported cases acquired in Egypt analyzed in isolates from ten Nile river cruise passengers.

Arashiro et al. [50] added a case report of a COVID-19 and Legionella co-infection in an 80-year-old Japanese male person who returned from a Nile cruise from 28 February –1 March 2020, who developed symptoms when returning to Japan.

### 3.3. COVID-19 on Navy Vessels

Three studies were identified dealing with SARS-CoV-2 infections on navy vessels referring to the USS Theodore Roosevelt, a navy aircraft carrier, and the USS Kidd, a navy guided missile destroyer [69,70,71]. The USS Theodore Roosevelt accommodates a crew of about 4985 persons, three of which were tested SARS-CoV-2 positive on 23 March 2020. After arriving at Guam’s harbor on 27 March 2020 and transferred to pier-side on 3 April 2020, when it also became known that 114 people were tested positive (one-third of the sailors who were tested). Therefore, a hospital was established in a tent to maintain medical treatment for the crew who were put under quarantine for two weeks in neighboring hotels or the Naval Base Guam (4232 crew members were transferred by 4 April 2020). To preserve the ships safety and carry out disinfection, 700 crew members stayed on board, were replaced after three weeks and put under quarantine in community hotels. Overall, 1102 of the approximate 4985 crew members were tested positive for SARS-CoV-2 (22% of the crew members within five weeks, including two dozen hospitalized crew members, and one death) [69]. Other studies showed that 736 had a confirmed infection of SARS-CoV-2, of which 590 crew members (80.2%) were symptomatic and 146 (19.8%) remained asymptomatic [71].

Payne et al. [70] gained additional insight into a convenience sample of 382 young adults experiencing the above reported outbreak on the USS Theodore Roosevelt. The presence of reactive antibodies were described for 60% and neutralizing antibodies were reported for 59% of those (overall indication of a current or previous SARS-CoV-2 infection by means of a positive PCR test result or a reactive antibody result for 238 individuals and no evidence of SARS-CoV-2 infection for 144 individuals). About 20% were examined as asymptomatic and two crew members (0.8%) were hospitalized. Public health measures, like the introduction of face coverings and social distancing had a positive impact on the risk for infection [70].

The second navy vessel (USS Kidd) disposed a capacity of 350 crew members [69]. It started its voyage in Hawaii in late March. The ship was detached from the USS Theodore Roosevelt while it was anchored pier-side in Guam during the SARS-CoV-2 outbreak. In late April, an outbreak of SARS-CoV-2 was reported while it was placed at the Pacific coast of South America. One crew member reported COVID-19 specific symptoms on 22 April 2020 wherefore he was transferred to the military hospital in San Antonio, Texas. One day later the COVID-19 diagnosis was confirmed. On 28 April 2020, after arriving in San Diego, a two-month process of disinfection, isolation, testing and treating measures was introduced. On 30 April 2020, 78 of approximately 300 tested crew members were evaluated as positive which represents about 25%. Palafox et al. [69] summarized that overall, 26 navy vessels reported COVID-19 outbreaks (1366 COVID-19 infections in early May 2020).

### 3.4. COVID-19 on Cargo Ships

Only one report of a SARS-CoV-2 infection on a cargo ship was identified within the time span of the review [72]. The Danish ship Gjertrud Maersk ran into port of Zhoushan on 17 March 2020 equipped with a crew of 22 members. One day later, a poor health condition of some crew members was reported to the local authorities by the ship’s agent. On 20 March 2020, an inspection took place and seven crew members were identified as concerned. While six days later the affected crew members were admitted to a hospital, the remaining ones were placed under medical observation on the ship. In the further course, five infections with SARS-CoV-2 were confirmed (all Filipino males, aged between 23 and 50 (mean age 37.8) and boarded in Hong Kong on 27 February 2020). Three of the five crew members who were tested positive for SARS-CoV-2 had symptoms, whereas the other 15 persons on board reported a good health status. The ship was no longer able to call at various ports planned such as Ningbo, Shanghai, Xiamen, Colombo, Felixstowe or Rotterdam, because it first had to be disinfected as a consequence. Likewise, the Chinese Ministry of Transport collaborated with other local authorities and evaluated e.g., challenges regarding crew rotation [72].

### 3.5. Problems Related to Identified Outbreaks on Ships Sailing Worldwide

When managing a SARS-CoV-2 outbreak with a high proportion of asymptomatic individuals on cruise, cargo or navy ships in a confined setting [54,62], several problems were reported. For cruise ships, not only a unique environment consisting of senior passengers with varying comorbidities was described [40,44,54,73], but also cultural diversity and language barriers [40,46]. Taking into account available resources, several authors illustrated limited equipment including beds, medication, testing capacities, negative pressure chambers and isolations facilities for more than 3000 concerned persons [40,43,48,64], wherefore sharing a cabin was necessary and transmission within the cabins continued after quarantine [44,49]. Moreover, when being confronted with limited medical resources for the inspection of several thousand individuals in a short period of time, it was outlined that the selection of suitable individuals receiving a test may result in allocation issues in case of an emergency [43].

Consequently, crew members were obligated to continue ships operations as well as daily services including food, sanitation and waste disposal [40,43,46,47,49]. Likewise, they needed to acquire medical professionalism and perform like voluntary medical staff. Nakazawa et al. [43] described that all persons onboard (crew members as well as passengers) on isolated ships were exposed to severe stress. Additionally, crew members and passengers remained on board during different subsequent voyages providing a potential transmission of SARS-CoV-2 among different ship populations as shown for the Grand Princess [42].

Regarding transportation issues and lacking onshore quarantine facilities, Japanese authorities classified a direct disembarkation and a land-based quarantine as not feasible referring to the Diamond Princess [40]. Yamahata and Shibata [47] specified challenges regarding the security of traffic lines, coordination of transportation means and also transporting processes on the ship. Additionally, an elevated risk of deterioration of the individuals’ medical condition needs to be considered since it was reported that about 10% who were transported from the ship developed mild chest pain during the six hour transportation as a result of a pneumonia and 10–20% of them deteriorated rapidly needing intubation within 24 h [47]. Overall, the transfer of SARS-CoV-2 positive passengers by means of helicopters was not allowed because protection of patients could not be ensured during air transport. Therefore, all patients with COVID-19 needed to be transferred by land [64]. Initially, when several family members received a positive test result, it was specified that they could not be admitted to the same hospital because of a limited amount of isolation beds. Other challenges aroused from the fact that positive and negative tested family members were separated, because it was challenging for passengers to leave the ship because of the measures taken, which often resulted in stress and a lack of understanding. Therefore, the DMAT chose to transfer patients with their families (independent of the infection status) to the same hospital by means of general hospital beds [64].

When returning back home after a cruise trip, challenges in the context of contact tracing and the use of different transportation means were described [74]. During the early phase of the pandemic, 445 contacts were monitored in the Northern Territory of Australia. A total 46 close contacts from cruise ships were monitored whereof 2 (4.3%; 95% CI 0.5–14.8%) became cases. However, the two cases had boarded a flight while being infectious after disembarking a cruise ship (not specified) with reported onboard transmission. A transmission of the virus was not observed to 21 close aircraft contacts and one house-hold contact of the two cases [74].

Overall, when referring to communication issues, complex command lines of different participating parties were reported [40] which needed to be coordinated including national and local authorities and public health departments, foreign authorities or embassies, hospitals, laboratories, and the shipping companies [42]. Another challenge was caused by the refusal of entry, e.g., into Japan because of national safety reasons due to the COVID-19 pandemic as shown in the example of MS Westerdam without knowing which port to call at [43].

On navy ships, problems resulted due to limited access to health care, limited personnel capacities and more crowded living conditions in which it was difficult to implement social distancing measures. Therefore, support was sought within the Guam community providing isolation facilities in hotels and the community while a medical unit on the navy base and a field hospital were set up [69].

## 4. Discussion

The main goal of the review was to summarize the current state of research on outbreaks of SARS-CoV-2 on ships sailing worldwide taking into account outbreak management techniques, occurred problems as well as recommendations for action.

### 4.1. Identified Outbreaks on Cruise Ships Sailing Worldwide

Overall, it was shown that cruise ships are closed environments with confined public rooms and accommodations, shared sanitary facilities, joint water and food provisions accommodating a high population density of persons from different countries [75]. Other challenges resulted from the displayed age structure on the cruise ships accommodating a high number of older and often above average multi-morbid people which were exposed to an increased risk of complications due to COVID-19 [41,42]. Furthermore, varying service expectations and responses to authoritarian rules of conduct during quarantine could be added [75]. Hence, a comprehensive outbreak management on ships was presented as challenging, especially when handling the quarantine/isolation of infected persons as shown in the example of the Diamond Princess.

On the Diamond Princess, the largest outbreak of SARS-CoV-2 in terms of the proportion affected among a defined population was described during the initial phase of the pandemic in February 2020 beside mainland China. The median incubation time in current studies was described as five to six days [27] which made the management of SARS-CoV-2 especially problematic for cruise ships. The initial intention of the quarantine of the Diamond Princess was to protect the general population by isolating passengers of the ship. However, on-board transmission occurred not only during e.g., mass gatherings before the implemented quarantine, it also proceeded when passengers were isolated/quarantined in their cabins when being exposed to the virus beforehand [42,46]. A corresponding elevated contact rate of guest to guest and among crew members compared to those from guest to crew, as a possible result of high transmission activities within shared cabins was presented (although influenced by a delayed testing of crew members) [32]. Therefore, triage based only on the persons’ symptoms without considering their cabin status was presented as insufficient to stop transmission [44].

Considering the number of cases only—apart from other ethical issues posed by the quarantine practice—the implementation of on-board isolation and quarantine could be questioned by results from mathematical modelling e.g., conducted by Röcklöv et al. [32]. The authors estimated that an early evacuation of all passengers would have resulted in 76 infected persons within their incubation time [32]. Sawano et al. [76] compared the rapid transmission of the virus on cruise ships even to an incubator due to the closed environment. Considerable ethical issues were raised by Dahl [75] who discussed about controversies not only for passengers but especially for crew members on board. In order to secure the daily services on board, not only the regular daily services were performed but also additional tasks, without being trained sufficiently as applied to many crew members. Those services had to be conducted under a greater risk of transmission of SARS-CoV-2 when permanently and closely interacting with passengers and/or infectious colleagues during or after work. Thus, on the one hand, the general population ashore could be protected, but on the other hand, the situation under quarantine was hardly bearable for everyone involved. Both passengers and crew members reported helplessness, anxiety, as well as physical and mental exhaustion [75].

Likewise, a high proportion of asymptomatic infections at the time of testing was described in the literature. Available studies with a sample size of more than 500 participants underline an asymptomatic course of the disease between 46.5% und 58.9% of confirmed cases [42,47,48,49]. Compared to more recent results from a systematic review and meta-analysis it was assumed that in 79 studies conducted in varying settings beside the maritime sector, 20% (95% confidence interval [CI] 17–25%) of those infected with SARS-CoV-2 were presented as asymptomatic. Additionally, a transmission of SARS-CoV-2 among contacts of people remaining asymptomatic was less likely compared to those with a symptomatic course of infection (relative risk 0.35, 95% CI 0.10–1.27) [77]. Nevertheless, given the fact that test capacities are limited or varying at each port to call at and many SARS-CoV-2 infections proceed asymptomatically, hygiene measures on ships like hand hygiene, wearing face coverings, and social distancing should be strengthened in infection prevention on any type of ship [78,79,80]. Moreover, in the future, the cruise industry should consider rethinking the design of ventilation systems and equip cruise ships accordingly [81]. Especially because heating, ventilation and air-conditioning (HVAC) systems might have an additional impact in decreasing the airborne transmission of SARS-CoV 2 [82].

Overall, scientific reports or publications are far from being written for all outbreaks of SARS-CoV-2 onboard cruise ships. Brief information on other outbreaks was available referring to Costa Magica, Costa Favolosa, Celebrity Eclipse, Disney Wonder, Holland America Zaandam or the Coral Princess and were summarized in the “Modification and Extension of No Sail Order and Other Measures Related to Operations” provided by the U.S. Department of Health and Human Services and the CDC [33]. On the first two ships, a total of 88 crew members with relevant symptoms were identified. Due to a need for urgent treatment, four persons had to be transported from the ship on the Costa Magica and seven persons on the Costa Favolosa in cooperation with the responsible authorities. On the Zaandam, 250 guests and crew members with COVID-19 specific symptoms were described, 76 of whom remained symptomatic and four died (one of them independently of COVID-19). An emergency treatment of a passenger due to COVID-19 was also reported on the Celebrity Eclipse as well as four crew members who fell ill. Besides the Celebrity Eclipse, four infections were also reported among crew members on the Disney Wonder, two of which had to be taken to hospital. Three guests from a previous voyage were also evaluated as positive [33]. On the Coral Princess, 12 cases were confirmed on board, seven among passengers and five among crew members and 43 suspected cases with respiratory symptoms in April 2020. Four persons had to be ventilated [33]. Furthermore, flight-associated transmission of SARS-CoV-2 was also reported among cruise ship passengers who disembarked from the Ruby Princess in Sydney with a retrospectively identified outbreak of SARS-CoV-2 [83]. For this reason, a comprehensive reappraisal should be carried out in scientific studies in order to guarantee a safe re-start for the cruise industry.

Further challenges aroused due to docking restrictions for cruise ships. Not only the MS Westerdam was refused entry to Japan because of national safety reasons [42], but other cruise ships had encountered similar problems. The World Dream was put under quarantine in Hong Kong after arriving on 5 February 2020 carrying three passengers with respiratory symptoms. Beforehand, Taiwanese authorities denied entry on 4 February 2020 [84]. Similar events took place on the Zaandam, which was rejected in mid-March 2020 in Chile, followed by a delayed voyage through the Panama Canal and a negotiation to dock at Port Everglades, United States [85]. On 15 March 2020, Australia excluded cruise ships entering from foreign ports [86]. In Canada, ships were prohibited from entering territorial areas until autumn 2020 [87]. Since many of the crew members were forbidden to disembark or could not fly back to their home country, thousands had to remain on board and were even not paid. Fears, social isolation, a considerable stressful life up to suicide were reported in the further course [87]. The procedure of refusing entries can be critically questioned referring to the definition of points of entry based on the International Health Regulations (IHR) [88], which provides a framework for countries for prevention, preparedness and response to public health risks. To protect travelers and the general population, states parties are obligated to sustain certain capacities at designated ports, airports or ground crossings to identify health risks at source, to be able to act in case of an emergency and to derive recommendations for action [88]. However, controversies arose during the COVID-19 pandemic concerning the principle of free pratique (permission for a ship to come into port, dis- or embark, discharge or load cargo or stores) [88]. In case of infections or contaminations on board, measures for disinfection, decontamination, disinsection or derating should take place to prevent its further spread. Additionally, countries can take supplementary health measures, given that such measures are based on available scientific evidence or advice from the World Health Organization (WHO, Geneva, Switzerland) [88]. As a result, decisions on health risks of concerned persons require sufficient scientific evidence, otherwise further interventions introduced by an individual country will be refuted by other parties [81]. Further discussions on the legal controversies and inconsistencies of international norms and national regulations can be assessed from Zhang and Wang [81 who claimed for a strengthened international cooperation based on rules including the exchange of information and management practices, more supervisory instruments, an amplification of key rules and distributions of obligations of different parties like port and flag states, national states, and companies.

### 4.2. Identified Outbreaks on Navy Vessels Sailing Worldwide

In addition to numerous events and challenges on cruise ships, results on navy vessels were reported. In November 2020, more recent results beside the time-span of the review were underlined by Kasper et al. [89] including further approaches for the described outbreak of the U.S.S. Theodore Roosevelt in late March, 2020. 1271 crew members (26.6%) were tested positive for SARS-CoV-2 including 1000 infections five weeks after the first confirmed infection. Likewise, the challenge posed by an asymptomatic disease progression became evident: for those crew members with a confirmed diagnosis 76.9% (*n* = 978) reported no symptoms when being tested. 55.0% were presented with symptoms in the further course. The studied population was presented as a young and healthy population with a mean age of 27 years. However, compared to cruise or cargo ships the working conditions on navy vessels were described as generally more confined, where crew members sleep in open bays, confined bunks, work in crowded areas or encounter each other in gyms or galleys [89]. Other studies by Letizia et al. [90] analyzed SARS-CoV-2 infections among U.S. Marine Corps recruits during a two-week quarantine at home and an additional two-week quarantine at a closed college campus under supervision including the use of masks, social distancing, as well as observing of temperature and symptoms. Of a total of 1848 participants, 16 (0.9%) were tested positive for SARS-CoV-2 after two days on the campus, including 15 asymptomatic courses. A total 35 infections (1.9%) were detected on day 7 or on day 14 (five of the 51 positive tested participants (9.8%) had symptoms before testing). Additional results of 36 genome analysis indicated six clusters among 18 participants including a transmission among roommates and recruits working in the same platoon [90].

### 4.3. Identified Outbreaks on Cargo Ships Sailing Worldwide

Only one report in the context of SARS-CoV-2 on cargo ships could be identified within the framework of the review criteria as reported by Dai et al. [72]. Two additional recent case reports of SARS-CoV-2 outbreaks on cargo ships outside the time-span of the review were published in January 2021 from Hamburg [91]. In each case, the port health authority in Hamburg was informed of symptomatic crew members (isolated on the chamber) in the spring and summer of 2020, respectively, either via the ship’s agent or the vessel traffic service center. Both ships were previously in a high-risk area without shore leave and external persons were contacted considering physical distance and the use of masks. On the first ship, a total of four crew members were tested positive for SARS-CoV-2 (one person asymptomatic) and on the second ship, six confirmed infections of SARS-CoV-2 were reported (a total of 26 crew members on each ship). For the first ship, there was close cooperation with the German Seamen’s Mission regarding land-based options for accommodation during quarantine, which also provided emergency psychosocial care. Some crew members of the second ship were accommodated in a residential accommodation. A minimum amount of crew number maintaining the ships safety was defined for each ship (16 and 15 persons, respectively), wherefore the remaining crew members could be isolated/quarantined ashore. Further problems aroused due to secured access routes to the ship, which could be ensured in cooperation with the harbor master by means of a mobile gangway [91].

Other investigations without confirmed infections of SARS-CoV-2 were presented by Fernandes et al. [92] reporting about a Chinese cargo ship running into port in February 2020 in Brazil at the Port of Santos with feverish crew members having respiratory symptoms. All 25 crew members were examined among whom no suspect cases could be identified. Again, collaboration between different organizations during surveillance was reported as crucial when investigating the outbreak [92]. Further challenges emerged in the cargo shipping sector due to crew change practices: after many months on board, the crew had to extend their service in some cases because they could not be replaced or fly home due to the prevailing travel restrictions [87]. According to a joint action statement of the UN, about 150,000 seafarers a month will need international flights due to crew changes [93]. Doumbia-Henry [87] described that in June 2020, only 30% of governments permitted crew changes, representing a main risk for the safety and well-being of seafarers as well as for the ships safety. Such pandemic-related conditions can cause additional fears and worries for many crew members, e.g., if the travel time is extended considerably and indefinitely due to lacking replacement possibilities [87].

### 4.4. Recommendations

Based on the current state of research several recommendations can be derived for enabling a safe start of cruise ship operations supplemented by several other organizations like the WHO [80,94] the ECDC [78] or projects like the EU Healthy Gateways Joint Action [79]. Generally, the responsibility for controlling the standards on board the ship is located by the flag state, while the states that the ship visits (port states) are in charge for defining the requirements for a ship when arriving its territorial waters [78].

First and foremost, before entering a port, the master of the ship is responsible for reporting about any suspected case of COVID-19 to the local authorities based on the IHR [88]. Therefore, port states should provide certain resources to maintain a suitable public health emergency response based on a comprehensive and up-to-date contingency plan, which should be communicated to operators including information on contact tracing, isolation practices of contact persons, procedures for disembarking or transportation to local hospitals [78].

#### 4.4.1. Recommendations for Cruise Ships

In accordance with the recommendations of the WHO, ECDC and the Healthy Gateways Joint Action [78,79,80], a written ship management plan for handling outbreaks of SARS-CoV-2 should be prepared. Topics like how to define and manage suspected SARS-CoV-2 infections and related close contacts, locations where to isolate suspect cases until they are able to disembark, a communication structure displaying different involved departments, the use of Personal Protective Equipment (PPE), practices for disinfection (according to each category, frequency of cleaning, appropriate products and techniques), waste management, performance of daily services and dealing with Passenger/Crew Locator Forms (PLF) should be included. Preparedness for receiving medical treatment ashore, quarantine facilities (e.g., hotels) for close contacts at the home port and organizing repatriations or crew changes if needed should by checked and implemented in the management plan by cruise ship operators [64,79]. Therefore, at the home ports or at least at one of the ports during the journey, collaborations with airports providing international flights are recommended [79].

Moreover, the risk for introducing SARS-CoV-2 onto any type of ship should be tackled by providing information from pre-boarding to disembarkation addressing the main symptoms of COVID-19, consequences for vulnerable groups, possibilities for prevention, health screenings and protocols concerning repatriation or disembarkation during an outbreak by means of leaflets, posters or communication strategies [78,79,80]. Moreover, SARS-CoV-2 specific information should be displayed on board providing instructions on social distancing, maximum capacities and the use of PPE. These measures should also be applied during community visits [78]. Additional information should be collected before starting and during a journey by cruise ship operations, keeping an eye on epidemiological developments worldwide and at the planned destinations [79], so that disembarkation at tourist sites with high virus circulation can be dispensed. As described in the literature, further challenges resulted e.g., from the age structure on cruise ships [40,54,73]. Therefore, passengers and crew belonging to high risk groups based on their age or medical conditions should receive special attention and be instructed to seek medical advice beforehand [79].

The current practice of social distancing and hygiene rules like wearing appropriate face-masks cleaning hands with water and soap or alcohol-based hand-rub solutions as well as the coughing and sneezing etiquette are particularly important [79,95]. Suitable actions for all situations or events where contact amongst passengers is likely should be reviewed if social distancing can be ensured and overcrowding can be reduced. Therefore, floor markings highlighting the recommended distance could be supplemented. In addition, reducing the passenger manifest should also be considered. Further protective barriers can be introduced as well to maintain safe interactions between crew members and passengers [78] and supplemented with future research on the design of ventilation systems [81].

The importance of non-pharmaceutical interventions like wearing a face covering or avoiding common areas should be reinforced as well [70,78,79]. The use of face masks is recommended when social distancing cannot be guaranteed or when the risk of infection is increased (e.g., indoors or in the wardroom) [78]. Ship operators should evaluate events or areas where the use of PPE is required and also define type and certification due to the protection of all persons on board. The use of PPE in the passenger terminals needs to be agreed with concerned Port State authorities [78]. The contact to non-ship personnel including inspectors, pilots or supply contractors should be reduced to the absolutely necessary and documented by a protocol leader. Additionally, alcohol-based hand disinfectants, or comparable, should be implemented in frequently visited areas, e.g., entrances, security areas, restaurants, lifts, corridors, cabins, sanitary and working spatializes or changing rooms [78].

Overall, health screenings should be considered from pre-boarding to disembarkation, including also re-embarkation after visits, tours or trips [78,80]. Current information on transmission risks should be collected and communicated to all persons on board and supplemented by close contact with the local public health authorities about local regulations [78,80]. Not only cruise operators should implement the described public health interventions, also excursion or tour providers or those maintaining external services should provide the same standards [78]. Passengers reporting about COVID-19 specific symptoms or about close contacts with confirmed infections will be refused to board a ship before starting a journey [79]. However, screening measures may not recognize mild or asymptomatic courses, incubating travelers or those concealing symptoms, who would still have access to the ship [79].

When referring to test strategies and the availability of tests, practices and requirements in different countries or shipping companies vary. At the time of writing, the PCR test is represented as the most accurate one to confirm SARS-CoV-2, though it is not always accessible besides health care settings [95]. Noteworthy, the latter circumstances are gradually, although partly, changing, since (even PCR) testing capacities on board are offered for SARS-CoV-2 infections [96]. Cruise ship operators e.g., in the U.S., must provide on board testing capabilities (in collaboration with the CDC) to be able to test all symptomatic persons including crew and future passengers and their close contacts for SARS-CoV-2 [97]. Overall, suitable laboratory testing capacities for SARS-CoV-2 should be ensured beforehand including on board facilities or by means of collaboration with laboratories ashore to provide PCR tests [79]. Other complementary strategies could be implemented e.g., by means of Antigen rapid tests. However, the results must be interpreted critically due to the limited sensitivity [98].

When identifying a suspect COVID-19 case, further information on disembarkation processes can be accessed from the WHO [80] including recommendations to reduce the risk of transmission for other persons. The implementation of an on board isolation and quarantine of passengers and crew members of a whole ship should be avoided due to results of mathematical modelling [32], wherefore an early evacuation of the ship should be sought. Contact tracing should start right after identifying a suspect case (definitions accessible from the WHO [80] or the ECDC [78]) independently of laboratory confirmation. When analyzing close contacts, different factors like shared cabins, group travelling, onshore activities, visits in restaurants or bars, activities on board (gym, theatre, cinema etc.), deck location should be comprehensively reviewed [44,67,80]. Therefore, several documents can be evaluated including the ship manifest or schematics, cabin lists, activities or dining reservation lists, medical logs, crew members assigned to each cabin and associated shifts and also demographic characteristics of passengers as a result of the age structure on a ship [80]. The PLF/crew locator form covers a minimum of data on each passenger [80]. Additionally, if applicable the use of mobile contact tracing apps can be supplemented [78]. In general, crew members should be educated to report about COVID-19 specific symptoms to supervisors or managers/ships officer in charge, not performing regular duties in case of symptoms, followed by self-isolation and suitable PPE without fear of personal disadvantages beyond the restrictions [79,80].

As shown by Jimi and Hashimoto [40] and Moriarty et al. [42], complex command lines of different participating organizations were involved during the management of SARS-CoV-2 on cruise ships [40,42]. Therefore, it was suggested to implement a communication strategy including predefined messages, means of communication and timing at different stages during ticketing, at the terminal, during a journey on board or in case of an outbreak of SARS-CoV-2 [79]. According to the ECDC [78], health authorities dealing with occupational safety and health issues, port state authorities handling international issues on the safety, security and environmental perspective, port authorities and terminals managing logistic operations as well as those dealing with transport, civil protection, home affairs and immigration should be included in planning processes. However, differences may occur when implementing a ship management plan at different ports or states (all the tasks pooled in one authority vs. distribution amongst different authorities) [78]. Therefore, to facilitate communication and coordination it is recommended that before starting a voyage ship management plans should be exchanged between the company and the port state/port authority/terminal operators and vice versa, ensuring compatibility between the plans, especially when cooperation is needed (e.g., during (dis- or re-)embarkation, when involving passenger terminals, after visits onshore, for crew change and repatriation, during the implementation of test strategies, management of suspected cases of COVID-19 and its contacts, waste disposal or other relevant efforts) [78]. Likewise, each port state should implement contact points for cruise lines enabling direct communication lines when preparing a re-starting of cruise operations (preferably a single contact point per port state to facilitate internal coordination) [78].

#### 4.4.2. Recommendations for Cargo and Navy Ships

Similar suggestions apply to cargo and navy ships without contact to passengers as to cruise ships e.g., in the area of disinfection of surfaces, education about symptoms, the implementation of hygiene and distance regulations or the provision of PPE (e.g., disposable gloves, long-sleeved and impermeable protective clothing, protective goggles or face shields (shields, visors), medical face masks and FFP2/FFP3 masks (or (K)N95)) [69,94,99]. Though, it should be kept in mind that on navy ships, more crowded living conditions were described, wherefore strict quarantine and isolation practices as well as limited shore leave must be implemented [69].

Further organizational measures can be considered, such as the establishment of hygiene record keepers when in contact with external persons, documenting contact details, period of presence and hygiene rules applied. In general, crew members should only have contact with port personnel for essential operational and administrative reasons maintaining a continued operation and supply of the ship [94]. To provide a basis for infection control, crew members’ knowledge of COVID-19 should be strengthened in terms of infection prevention, but interventions are still needed to underline awareness of the risks and engagement in containment of SARS-CoV-2 [100]. In general, all crew members are obliged to self-monitor their health status. In case of an onset of COVID-19 specific symptoms, they should report themselves to the persons responsible on board followed by a subsequent reporting to local authorities from recent and following ports of the ship’s itinerary [95]. Aggravatingly, the cargo shipping sector faces high levels of economic pressure, because ships are subject to strict schedules and short lengths of port stays [91].

Onboard, the World Health Organization (WHO) [94] defines four zones on a ship including potentially contaminated zones (1) where suspected cases can be isolated, zones for interaction of crew members (such as mess rooms, the bridge, control rooms, or shared cabins) (2), zones for encountering with shore personnel (3) and such where no interaction takes place (like single cabins) (4) [94]. In the first zone of contamination, all persons entering the isolation area should wear a medical mask and apply standard precautions as described in WHO’s guidance for infection control [94]. In Zones Two and Three, crew members must consider recommendations of social distancing, taking also the arrangement of communal areas, seats and workstations into account. In case distancing cannot be secured, persons in charge like the master or skipper should evaluate if a continuation is necessary. When doing so, additional actions like the use of masks should be recommended. Additionally, frequently used routes onboard should be taken while maintaining the necessary distance or external stairways/escape routes and walkways if those are permitted and safe [94,101]. If possible, work equipment should be used on a person-by-person basis or disinfected regularly before being handed [99,101]. In general, information exchange, e.g., during a handover, should be done in the open, in writing, or by telephone.

Furthermore, pre-boarding screening is advised for newly enrolling crew members to identify any infected crew member or those who were exposed to infected individuals. Therefore, self-quarantine, arrival precautions, testing, and pre-boarding self-disclosure should be used [94]. According to the International Maritime Organization [101], shipping companies should ensure that crew members strictly fulfil locally or nationally isolation, quarantine and/or testing practices before leaving their country of residence and those required by relevant authorities in transit and destination countries where the crew change will be carried out. Therefore, crew members should be provided with relevant information on the company’s policies [101].

As described earlier, the use of rapid testing may still be considered on board to break chains of infection as a supplementing strategy. Currently COVID-19 vaccines are only available through national, government-run vaccination programs. However, e.g., the International Chamber of Shipping [102] is looking for ways for seafarers to obtain vaccines.

Regarding the definition of suspected cases and close contacts, it is recommended to define the following persons as suspected cases: those who shared the same cabin, those who had close contact, e.g., when eating together or sharing watch in an engine control room, those stayed in contact to a suspected or confirmed case, those who traveled together, those who cleaned the cabin of a suspected case, or medical caregivers [102]. In case of suspected cases, they should be placed in isolation in a designated area like a cabin without having contact to other crew members. Other crew members entering an isolation room are supposed to wear impermeable gowns, goggles, gloves and a medical mask [94]. Telephone or chat exchanges with isolated crew members should also be used to minimize in-person demands. Ideally, crew members should be isolated/quarantined in suitable quarantine facilities on shore. Experiences from the Port of Hamburg indicated that a minimum amount of crew members need to stay on board for maintaining the ships safety, wherefore not all crew members can be isolated/quarantined ashore. Furthermore, it was emphasized that access routes to ships in the port should be ensured at all times [91].

Overall, before calling a port, the Telemedical Maritime Assistance Service (TMAS) poses an essential supplement of seafarers’ health protection and provides support and advice to seafarers in case of illness, accidents, maritime emergencies or other incidents where medical advice is needed on board. The captain or a ship’s officer—a non-professional medical crew member—is responsible for medical care on board. Therefore, the TMAS is available 24 h a day for support, free of charge and is offered to all ships worldwide [103].

#### 4.4.3. Recommendations on Mental Health

On all types of ships, mental health was identified as a key issue during outbreak management (e.g., [43,87]). Psychosocial emergency care serve as an important component when dealing with catastrophic events. For instance the disaster psychiatric assistance team (DPAT) in Japan supported during the outbreak of COVID-19 on cruise ships [43]. Additionally, von Münster et al. [91] recommended with regard to the cargo shipping sector, to isolate persons with confirmed infections of SARS-CoV-2 in land bases facilities to not only reduce the risk of exposure for healthy crew members, but also for treating complicated courses and the provision of psychosocial care (e.g., in terms of panic attacks by isolated crew members on cargo ships). Furthermore, incomplete information and communication barriers can also increase uncertainty and the feeling of being at the mercy of others, and conflict situations can arise. Close psychosocial support for those affected is urgently needed. Worries and fears must be perceived and individual needs must be addressed [104].

To be able to react to the problem of mental health issues due to limited crew change practices in the cargo shipping sector, as an example, the German Seamen’s Mission had provided a chat platform on which seafarers can address their concerns, worries and needs to full-time employees of the German Seamen’s Missions [105]. In addition, crisis intervention teams were also provided free of charge by various organizations [87]. When it comes to crew changes, concerned Port States and authorities should ensure to facilitate crew changes and the repatriation of seafarers guaranteeing access to medical care and reasonable transport conditions [78]. To sum up, further research is needed that deals with the seafarers’ demands, strain and resources in order to provide suitable options for intervention.

#### 4.4.4. Recommendations on Training Practices

In general, training practices should be expanded for concerned employees including medical staff for sample collection and testing on board of a cruise ship, ship officers, crew members as well as external organizations who may have contact with passengers or crew members. In particular, the use of PPE and hygiene procedures should be trained and supplemented with special guidance for those who interact with others on a frequent level (e.g., during cleaning or security checks) [78,79]. Extra time to perform additional duties should be provided without impacting the performance of regular tasks or rest periods which could affect the wide-ranging safety of the ship as a result. Overall, measures for prevention, surveillance of SARS-CoV-2 and the subsequent response to an outbreak including the time of response, collaboration and communication issues, procedures and related equipment should be part of regular trainings like table-top exercises or drills [79]. One example in this context is represented by the model project ARMIHN-project (Adaptive Resilience Management in the Port) [106], which aims to improve resilience and capability in case of an outbreak of an infectious disease on ships or in the port. Within the study, all stakeholders within the rescue chain in the Port of Hamburg, Germany are included, damage scenarios due to infectious diseases on board of ships are used and a suitable adaptive emergency and communication concept with a corresponding training concept is developed and afterwards adapted for other ports.

Overall, the current pandemic needs to be seen as a universal challenge, which can only be tackled by mutual and worldwide collaboration [43]. In the future, transparency is required in terms of procedures, accountability and fast international cooperation [43], especially when being confronted with the spread of new virus variants, for example from South Africa (B.1.351), the Great Britain (B.1.1.7), or Brazil (P.1) which, according to initial studies, is associated with higher transmissibility [30]. Additional challenges result due to the allocation of vaccination through national, government-run vaccination programs [102].

### 4.5. Strengths and Limitations

A strength of the present review was the framework including a systematic approach using the PEO-criteria (population, exposition and outcome) by Khan et al. [35]. Thus, inclusion and exclusion criteria of search items were previously set. To ensure that the literature was searched comprehensively, four databases were included to identify eligible studies. In addition, a hand search by two researchers served as an adequate supplement to the previous systematic search. Afterwards, reference lists of all included studies were read through and analyzed. In addition, full text screening as well as titles and abstract screening were conducted by two researchers independently for higher eligibility, a moderate Cohens-Kappa (0.78) confirmed a substantial agreement between them (based on Landis and Koch [38]). Another strength of the review was the consideration of a broad spectrum of studies dealing with the maritime sector and the derivation of recommendations for practice.

However, several limitations should be noted. It is important to point out that not all outbreaks on all kind of ships were depicted in the literature at the time of writing compared to media attention. Moreover, additional outbreaks of SARS-CoV-2 might have been reported since July 2020 and therefore not covered by the systematic review. Furthermore, a varying methodological quality of included studies needs to be highlighted. When interpreting the results it should be considered that the included studies often referred to small sample sizes or related to single case studies of COVID-19 patients or mathematical assumptions by using cross-sectional data. Access to empirical data on board might be limited, e.g., because of possible testing bias (testing more passengers than crew members, see Röcklöv et al. [32]) and needs to be rechecked when all data is available. Overall, only very limited evidence was identified concerning the cargo shipping sector and navy vessels. All in all, these factors might affect the generalizability of our results on the maritime sector in general.

## 5. Conclusions

This review indicates that outbreaks of SARS-CoV-2 could be challenging in the maritime sector, not only on cruise, but also on cargo ships or navy vessels, although limited evidence has been identified for the latter two vessel types. Overall, the Diamond Princess was comprehensively researched as it was presented as the largest outbreak of SARS-CoV-2 during the initial phase of the pandemic in February 2020 beside mainland China. Challenges resulted from a high proportion of asymptomatic cases in a closed environment accommodating numerous senior passengers with varying comorbidities. Furthermore, limited equipment including beds, medication or testing capacities, were depicted as well as transportation issues, complex communication lines between different organizations and several challenges for crew members, who were obligated to continue the ships operations. Other challenges aroused because of docking restrictions due to national safety reasons. Recommendations were derived based on the literature and supplemented by those of the WHO, ECDC and the Healthy Gateways Joint Action.

## Figures and Tables

**Table 1:** Inclusion and exclusion criteria.

	Inclusion Criteria	Exclusion Criteria
Population	Population on board of ships sailing worldwide	No reference to maritime context
Exposure	SARS-CoV-2	Exposure to other pathogens
Outcome	Reports of seroprevalences of SARS-CoV-2 antibodies or clinically/laboratory confirmed cases or outbreaks of SARS-CoV-2 infections on board ships	No confirmed SARS-CoV-2 infections on board.

**Table 2:** Characteristics of the included studies in the systematic review.

	*n*	%
Country of port called		
Japan	27	69.2
U.S.A	5	12.8
Egypt	3	7.7
Cambodia	1	2.6
China	1	2.6
unknown	2	5.1
Setting		
Cruise ship	33	89.2
Navy vessel	3	8.1
Cargo ship	1	2.7

## Supplementary Materials

The following are available online at https://www.mdpi.com/article/10.3390/ijerph18105195/s1, Table S1: Background information of included studies.
