# Supplementary material for: Systematic Review on Outbreaks of SARS-CoV-2 on Cruise, Navy and Cargo Ships
Source: Int J Environ Res Public Health. 2021 May 13;18(10):5195. doi: 10.3390/ijerph18105195 (PMC8153346; doi:10.3390/ijerph18105195)
Supplement: Supplementary file 1 [file ijerph-18-05195-s001.zip › ijerph-1176450-supplementary.pdf]

Supplementary Material

Systematic review on outbreaks of SARS-CoV-2 on cruise, navy and cargo ships

Ann-Christin Kordsmeyer[1*†], Natascha Mojtahedzadeh[1†], Jan Heidrich[1], Kristina Militzer[1], Thomas von Münster[1], Lukas Belz[1], Hans-Joachim Jensen[1], Sinan Bakir[2], Esther Henning[2], Julian Heuser[2], Angelina Klein[2], Nadine Sproessel[2], Axel Ekkernkamp[2], Lena Ehlers[3], Jens de Boer[3], Scarlett Kleine-Kampmann[3], Martin Dirksen-Fischer[3], Anita Plenge-Bönig[3], Volker Harth[1], Marcus Oldenburg[1]

[1] Institute for Occupational and Maritime Medicine (ZfAM), University Medical Center Hamburg-Eppendorf (UKE), 20459 Hamburg, Germany; n.mojtahedzadeh@uke.de (N.M.); jan.heidrich@justiz.hamburg.de (J.H.); kristina.militzer@justiz.hamburg.de (K.M.); thomas.vonmuenster@justiz.hamburg.de (T.v.M.); lukas.belz@justiz.hamburg.de (L.B.); hans_joachim-jensen@t-online.de (H.-J.J.); harth@uke.de (V.H.); marcus.oldenburg@justiz.hamburg.de (M.O.)

[2] Department of Trauma and Reconstructive Surgery and Rehabilitative Medicine, Medical University Greifswald, 17475 Greifswald, Germany; sinan.bakir@uni-greifswald.de (S.B.); Esther.Henning@med.uni-greifswald.de (E.H.); Julian.Heuser@med.uni-greifswald.de; (J.H.); ak166297@uni-greifswald.de (A.K.); Nadine.Sproessel@med.uni-greifswald.de (N.S.); traumato@uni-greifswald.de (A.E.)

[3] Hamburg Port Health Center (HPHC), Institute for Hygiene and Environment, 20537 Hamburg, Germany; lena.ehlers@hu.hamburg.de (L.E.); jens.deboer@hu.hamburg.de (J.d.B.); scarlett.kleine-kampmann@hu.hamburg.de (S.K.-K.); martin.dirksen-fischer@hu.hamburg.de (M.D.-F.)

[4] Infectious Diseases Surveillance Center, Institute for Hygiene and Environment, 20539 Hamburg, Germany; anita.plenge-boenig@hu.hamburg.de

* Correspondence: a.kordsmeyer@uke.de

† These authors contributed equally to this research.

.

Table S1. Background information of included studies.

Author, Year of Publication, Reference	Study Design; Method	Setting	Sample Size (*n*)	Population (crew /passengers)	Main Measurements	Aim of the Study	Main Results
Alvarado et al., 2020 [71]	Research Letter; standardized questionnaires	Navy vessels (USS Theodore Roosevelt)	Of 4,085 sailors, 736 had a confirmed infection of SARS-CoV-2	Crew members	Polymerase chain reaction (PCR)-Tests<br>Daily monitoring<br>Demographic data<br>Epidemiological data	• To highlight the results of an investigation of the USS Theodore Roosevelt SARS-CoV-2 outbreak and its management techniques	• 736 had a confirmed infection of SARS-CoV-2<br>• 590 sailors (80.2%) were classified as symptomatic and 146 as asymptomatic<br>• Cough was documented for 677 person-days (13.6%), cold-like symptoms for 483 person-days (9.7%), anosmia for 463 person-days (9.3%), headache for 438 person-days (8.8%), ageusia for 393 person-days (7.9%), and fever for 65 person-days (1.3%)<br>• 6 sailors were hospitalized, and 1 died
Anan et al., 2020 [64]	Field report	Cruise ship (Diamond Princess)	769 patients (696 with COVID-19) were transported to	Passengers and crew members	PCR-Tests<br>Information on transportation	• To illustrate management techniques and related problems when managing	• The Japan Disaster Medical Assistance Team (DMAT) transferred COVID-19 patients by

Author, Year of Publication, Reference	Study Design; Method	Setting	Sample Size (*n*)	Population (crew /passengers)	Main Measurements	Aim of the Study	Main Results
			a hospital			SARS-CoV-2 on the Diamond Princess	means of a novel classification system taking medical examinations of the passengers and selected hospitals into account
Arashiro, Furukawa & Nakamura, 2020 [65]	Research letter; Case report	Cruise ship (Diamond Princess)	2	Crew members	Clinical courses (illness and hospital days, symptom cessation day, symptoms like fever, cough, dry throat, rhinorrhea, congestion, medication) PCR-Tests	• To report about two cases of COVID-19 with mild upper respiratory symptoms	• Patient 1: described low-grade upper respiratory tract symptoms without fever<br>• Patient 2: had rapid symptom termination but persistent viral ribonucleic acid (RNA) was observed
Arashiro et al., 2020 [50]	Clinical Pearls; case report	Cruise ship (Nile river cruise)	1	Passenger	Clinical course (illness and hospital day, Arterial partial pressure of oxygen (PaO_2) Oxygen saturation ($SPO_{2:}$), symptoms like fever, malaise, cough, nausea, diarrhea,	To highlight a case and the importance of differential diagnosis during the current COVID-19 pandemic	Case report of an 80-year-old Japanese male passenger who was transferred into the hospital on 11 March with malaise, diarrhea and cough after visiting Egypt with a Nile cruise

Author, Year of Publication, Reference	Study Design; Method	Setting	Sample Size (*n*)	Population (crew /passengers)	Main Measurements	Aim of the Study	Main Results
					Legionella antigen, PCR-Test		
COVID-19, Australia, 2020 [73]	Epidemiological report; descriptive epidemiology	Cruise ships (not specified)	215 cases between 8 and 21 June, most cases were acquired locally compared to overseas-acquired cases	-	National trends, testing, source of acquisition, demographics of cases, severity, comorbidities, symptoms, populations of interest, school-aged children and older populations, public health response measures, international situation	• To report about notified cases of COVID-19 and associated deaths reported to the National Notifiable Diseases Surveillance System	• Of the oversea-related infections in older populations, cruise-ship-acquired infection represents 62% of the cases<br>• The highest rate of COVID-19 among people aged 65–79 years (Three-quarters of all cases associated with overseas travel, including outbreaks linked to cruise ships)
Dai et al., 2020 [72]	Letter to the editor; Case report	Cargo ship (Gjertrud Maersk)	5 cases of 22 crew members	crew members	Computer tomography (CT) scan, virus antibody test (Immuno-globulin M and G; IgM, IgG), nucleic acid tests, Symptoms like cough, fever or pharyngalia,	• To report about SARS-CoV-2 infections of cargo ship seafarers	• Five patients were tested positive for SARS-CoV-2<br>• All cases boarded in Hong Kong, China on 27 February and stayed on board<br>• Case 1: fever since 19 March<br>• Case 2: persistent cough and pharyngeal pain since 12 March

Author, Year of Publication, Reference	Study Design; Method	Setting	Sample Size (n)	Population (crew /passengers)	Main Measurements	Aim of the Study	Main Results
					boarding		• Case 3: mild cough on 10 March with slight white sputum<br>• The other two cases had no obvious clinical symptoms<br>• The five cases were transported to the hospital for isolation and treatment
Draper et al., 2020 [74]	Short report; public health follow-up of cases	Cruise ships (not specified)	28 cases of COVID-19 in the Northern Territory (NT) of Australia between 1 March and 30 April and 527 close contacts; 445 close contacts were located in the Northern Territory and 4 were tested positive after reporting about symptoms; 2 of 46 contacts who	Passengers	PCR-Tests, close contacts' quarantine addresses and the outcomes of daily monitoring	• To highlight the contact tracing methods in the NT during the period 1 March to 30 April 2020 and to describe the outcomes of those actions	• The first cases of COVID-19 notified in the NT were 8 passengers from the Diamond Princess who were repatriated to Australia<br>• Of the 46 close contacts from cruise Ships, 2 (4.3%; 95% CI 0.5–14.8%) became cases, after boarding a flight while infectious after disembarking a cruise ship with known on-board transmission (no transmission to

Author, Year of Publication, Reference	Study Design; Method	Setting	Sample Size (*n*)	Population (crew /passengers)	Main Measurements	Aim of the Study	Main Results
			were cruise ship passengers (4.3%, 95% CI 0.5–14.8%)				21 close aircraft contacts or 1 household contact)
Emery et al., 2020 [58]	Research article; Transmission model with asymptomatic and pre-symptomatic states	Cruise ship (Diamond Princess)	1304 estimated (1,198–1,416) infected individuals with SARS-CoV-2	Passengers and crew members	asymptom-atic and presymptomatic states, available data included: date of symptom onset for symptomatic individuals, the number of symptom-agnostic tests for each day, the date of positive tests for asymptomatic and pre-symptomatic individuals	• To shed light on asymptomatic infections of SARS-CoV-2 and their contribution to transmission	• The proportion of asymptomatic passengers was (74%), who may have contributed considerably to the spread of the disease (69% of asymptomatic individuals were presented as the source of all infections)<br>• The model estimated that 1,304 persons were infected (35% of the primary Diamond Princess population) whereof 53% of infections had not been detected, predominantly asymptomatic)
Expert task force for the COVID-19	Report;	Cruise ship (Diamond Princess)	2,666 passengers and 1,045	Passengers and crew members	PCR-Tests, information from the persons	• To gain insight into the	• 544 (20.4%) of the passengers were defined as case-

Author, Year of Publication, Reference	Study Design; Method	Setting	Sample Size (*n*)	Population (crew /passengers)	Main Measurements	Aim of the Study	Main Results
Cruise Ship Outbreak, 2020 [48]	descriptive and analytical statistics		crew members; 544 (20.4%) passengers were infected, and 143 (13.7%) crew members were infected		on the ship (age, sex, country of passport, cabin number, and classification as passenger or crew) symptom data (symptoms, onset date, and severe outcomes) time of specimen collection spatial distribution of confirmed cases attack rates by cabin occupancy	understanding of SARS-CoV-2 the epidemiology of an outbreak on a cruise ship was assessed	patients and 314 (57.7%) were asymptomatic (no clustering by deck or zone identified)<br>• Attack rates for passengers were highest in 4-person cabins<br>• 143 (13.7%) of crew members were defined as case-patients and 64 (44.8%) were asymptomatic (diffusive pattern across decks, large number of cases on deck 3)
Hung et al., 2020 [56]	Case series; SARS-CoV-2 shedding and seroconversion	Cruise ship (Diamond Princess)	215 adults, of whom nine (4%; 95% CI 2–8) had a confirmed SARS-CoV-2 infection	Passengers	PCR-Tests serum IgG and IgM against internal nucleoprotein and the surface spike receptor-binding protein (RBD) of SARS-CoV-2 at baseline and on days 4, 8, and 12 of quarantine	• To examine the shedding pattern of SARS-CoV-2 and patient antibody responses before and after the onset of symptoms	• Of 215 adults nine (4%; 95% CI 2–8) were positive for SARS-CoV-2 by PCR or serology and hospitalized<br>• Five patients (56%; 27–81) with ground-glass changes on CT were found to have higher anti-nucleoprotein-IgG optical density (OD) values on day 8 and

Author, Year of Publication, Reference	Study Design; Method	Setting	Sample Size (*n*)	Population (crew /passengers)	Main Measurements	Aim of the Study	Main Results
							12 and anti-RBD IgG OD value on day 12<br>• Six (67%; 35–88) patients remained asymptomatic<br>• Those patients with evidence of pneumonia have an increased antibody response
Inui et al., 2020 [39]	Research article; retrospective study	Cruise ship (Diamond Princess)	104 cases	Not specified	Chest CT medical records, demographic data, medical history, comorbidities, symptoms and signs	• To analyze chest CT findings of passengers from the Diamond Princess	• Of the 104 cases 76 were asymptomatic (73%), 41 (54%) had pneumonic changes on CT<br>• The other 28 cases (27%) were classified as asymptomatic (22 (79%) had abnormal CT findings)<br>• Asymptomatic cases were examined with more Ground-glass opacity (GGO) predominance over consolidation (83%) compared to symptomatic cases who were more likely to be found with a consolidation

Author, Year of Publication, Reference	Study Design; Method	Setting	Sample Size (n)	Population (crew /passengers)	Main Measurements	Aim of the Study	Main Results
							predominance over GGO (41%)<br>• Asymptomatic cases had lower CT severity scores compared to symptomatic cases
Jimi & Hashimoto, 2020 [40]	Report	Cruise ship (Diamond Princess)	712 (as of 17 March 2020) persons infected with SARS-CoV-2	Passengers and crew members	quarantine operation	• To underline quarantine operation on Diamond Princess and its principles	• During the quarantine period starting from 5 February 2020 a series of measures have been conducted under three principles: zero deaths among all persons on board, implementation of an infection control system and maintenance of health conditions among passengers and crew members)
Jorden et al., 2020 [66]	Morbidity and Mortality Weekly Report	Cruise ship (Gran Princess)	3 cases confirming cryptic circulation of the virus by early February	Not specified	community transmission	• To examine four lines of evidence providing insight into the timing of introduction and early transmission of SARS-CoV-2	• Three cases occurred indicating cryptic circulation of the virus by early February, one of them in an unidentified

Author, Year of Publication, Reference	Study Design; Method	Setting	Sample Size (*n*)	Population (crew /passengers)	Main Measurements	Aim of the Study	Main Results
							passenger or crew member aboard a Pacific cruise ship that left San Francisco on 11 February
Kakimoto et al., 2020 [41]	Morbidity and Mortality Weekly Report; Questionnaire of the cruise ship company and interviews (before disembarkation)	Cruise ship (Diamond Princess)	9 crew members	Crew members	Type of work Crew member cabin deck	• To gain insight into findings from the initial phase of the cruise ship investigation among crew members	• The earliest cases in crew members were reported in food service workers<br>• 15 of the 20 confirmed cases in crew members occurred among food service workers and 16 of the 20 cases occurred among persons with cabins on deck 3 (the deck of the food service workers)<br>• No systematic testing was implemented for crew members until 6 February 2020 only for those who reported about symptoms<br>• Three crew members reported subjective fever on 3

Author, Year of Publication, Reference	Study Design; Method	Setting	Sample Size (n)	Population (crew /passengers)	Main Measurements	Aim of the Study	Main Results
							February and 31 crew on 9 February
Kato et al., 2020 [54]	Note; Analysis of case report forms	Cruise ship (Diamond Princess)	70 patients	Not specified	Age, sex, race, underlying conditions, general status and vital signs, major symptoms, laboratory data, antibiotics, antiviral drugs, other supportive care, chest radiographs, CT findings	• To report the clinical conditions, treatment, and the clinical course of the patients positive for SARS-CoV-2	• Main symptoms: fever (64.3%), cough (54.3%), and general fatigue (24.3%)<br>• 61.4% had pneumonia<br>• Parameters associated with a pneumonia: higher body temperature, heart and respiratory rate, higher of lactate dehydrogenase (LDH), aspartate aminotransferase (AST), C-reactive protein (CRP) levels lower serum albumin level and lymphocyte count<br>• GGO reported for 97.7% with pneumonia<br>• Two patients died
Leung et al., 2020 [51]	Correspondence, case report	Cruise ship (Diamond Princess)	1 (index case)	Passenger	Clinical course of the index patient	• To highlight the diagnosis and management of the first passenger tested	• The 80-year-old Chinese resident took a flight from Hong Kong to Tokyo

Author, Year of Publication, Reference	Study Design; Method	Setting	Sample Size (*n*)	Population (crew /passengers)	Main Measurements	Aim of the Study	Main Results
						positive for SARS-CoV-2	on 17 January, embarked on the cruise ship in Yokohama, Tokyo on 20 January<br>• On 23 January, he reported mild dry cough<br>• After five days, the patient returned to Hong Kong<br>• On 29 January, he developed fever and went to the hospital; nasopharyngeal aspirate was evaluated positive for SARS-CoV-2 on 1 February
Liu et al., 2020 [59]	Research article; Mathematical modelling, contact network to simulate the two-stages outbreak of COVID-19	Cruise ship (Diamond Princess)	3711 persons on board including 2666 passengers and 1045 crew members, on 26 February, 705 SARS-CoV-2 infections were reported	Passengers and crew members	Basic reproductive number	• To develop a contact network and no-contact models to analyses the spread in heterogeneous populations and contact patterns	• During the early epidemic with intensive social contacts the transmissibility t was 0.026 and the basic reproductive number R0 was 6.94 (all people would be infected in one month)

Author, Year of Publication, Reference	Study Design; Method	Setting	Sample Size (*n*)	Population (crew /passengers)	Main Measurements	Aim of the Study	Main Results
							• After implementing the quarantine, t and R0 decreased to 0.0007 and 0.2
Miyamae et al., 2020 [57]	Short Communication; retrospective review of confirmed COVID-19 cases	Cruise ship (Diamond Princess)	23 cases	Not specified	age, sex, comorbidities, body temperature, peripheral oxygen saturation (SpO_2) on admission, highest recorded Body temperature and lowest SpO_2 during hospitalization, presence of symptoms, laboratory results (white blood cell count, lymphocyte count, LDH, blood urea nitrogen, serum creatinine, and CRP), imaging findings, Chest X-ray,	• To analyze the course of PCR assays and the duration of viral shedding in individuals with asymptomatic or mild infections	• The median duration of viral shedding was 19 days (range, 6–37 days) after initial viral detection<br>• Eight cases (35%) reported another positive PCR result after testing negative once

Author, Year of Publication, Reference	Study Design; Method	Setting	Sample Size (*n*)	Population (crew /passengers)	Main Measurements	Aim of the Study	Main Results
				PCR-tests			
Mizumoto & Chowell, 2020 [63]	Research article; Mathematical modelling using time-series incidence data by dates of symptoms onset	Cruise ship (Diamond Princess)	621 infected with SARS-CoV-2 of 3,711 passengers and crew members (as of 22 February 2020)	Passengers and crew members	Time-dependent reproduction number	• Characterization of temporal variation in the transmission potential of the COVID-19 outbreak aboard the Princess Cruises Ship	• Estimated reproduction number (~11) higher than mean estimates reported from community-level transmission dynamics in China and Singapore (range: 1.1.-7)<br>• Second outbreak aboard the Diamond Princess unlikely
Mizumoto et al., 2020 [60]	Rapid communication; Mathematical modelling	Cruise ship (Diamond Princess)	634 infected with SARS-CoV-2	Passengers and crew members	PCR-Tests	• To calculate the proportion of asymptomatic individuals on board of the Diamond Princess and their times of infections	• Asymptomatic proportion was estimated to be 17.9% (95% credible interval (CrI): 15.5–20.2%)<br>• Most infections were observed before the implementation of the quarantine
Moriarty et al., 2020 [42]	Morbidity and Mortality Weekly Report	Cruise ship (Diamond Princess and Grand Princess)	712 infected with SARS-CoV-2 of 3,711 persons on board of the	Passengers and crew members	Not specified	• To highlight the widespread transmission of SARS-CoV-2 and its consequences	• Of the Diamond Princess, 712 (19.2%) were tested positive for SARS-CoV-2 (among 3,711

Author, Year of Publication, Reference	Study Design; Method	Setting	Sample Size (n)	Population (crew /passengers)	Main Measurements	Aim of the Study	Main Results
			Diamond Princess; 78 infections with SARS-CoV-2 of 3571 people on board of the Grand Princess			• Describing public health responses to outbreaks on the Diamond Princess and Grand Princess	passengers and crew members)<br>• 331 (46.5%) were asymptomatic<br>• For the Grand Princess on 21 March, 78 (16.6%) were tested positive for SARS-CoV-2 (total of 469 persons with available test results)
Nakazawa et al., 2020 [43]	Research article; displaying chronological and ethical considerations	Cruise ship (Diamond Princess and MS Westerdam)	691 cases (as of 23 February 2020)	Passengers and crew members	Number of infected passengers and crew members	• To analyze the international coordination framework and the global ramifications of such outbreaks	• Ethical issues included aspects related to reasonable justification for isolation, the psychological fragility and quality of life during isolation, justice in a forced quarantine, and the improvement of control measures
National Institute of Infectious Diseases, Japan (NIID), 2020 [49]	Field Briefing: systematic collection of respiratory specimens	Cruise ship (Diamond Princess)	18 February, 531 confirmed cases; 3,711 individuals on board the Diamond	Passengers and crew members	Collection of respiratory specimens from all passengers by age group	• To report about the number of confirmed infections of passengers and crew members of	• There was a substantial transmission of SARS-CoV-2 before the implementation of quarantine on the Diamond

Author, Year of Publication, Reference	Study Design; Method	Setting	Sample Size (*n*)	Population (crew /passengers)	Main Measurements	Aim of the Study	Main Results
			Princess (2,666 passengers and 1,045 crew members)		via PCR-Tests, Epidemiological data: onset of symptoms, date of lab confirmation, and close contacts	the Diamond Princess	Princess on 5 February 2020<br>• The declined number of confirmed cases, implies an effective quarantine intervention for reducing transmission<br>• Some crew had to continue to perform essential duties for the functioning of the vessel and supplying passengers
Nishiura et al., 2020 [61]	Editorial; Statistical analiysis, Backcalculating the infection incidence of COVID-19	Cruise ship (Diamond Princess)	199 symptomatic cases (as of 24 February 2020)	Passengers and crew members	Statistical model with doubly interval-censored likelihood and right truncation with an exponential growth of cases using PCR results	• To understand the risk of infection over time	• The peak time of infection was observed during 2 and 4 February 2020, whereupon the incidence declined afterwards<br>• Without the quarantine from 5 February, the cumulative incidence with and without close contact was estimated to be

Author, Year of Publication, Reference	Study Design; Method	Setting	Sample Size (*n*)	Population (crew /passengers)	Main Measurements	Aim of the Study	Main Results
							1,373 (95% CI: 570, 2176) and 766 (95% CI: 587, 946) cases, respectively (102 and 47 cases observed, respectively)
Palafox et al., 2020 [69]	Viewpoint; Descriptive statistics	Navy Aircraft Carrier (USS Theodore Roosevelt); Navy guided missile destroyer (USS Kidd)	1,102 infections with SARS-CoV-2 of 4,985 people on board of the USS-Theodore Roosevelt; 78 of 300 tested crew members were positive of the of the USS Kidd	Crew members	Test-results (not specified)	• To present a framework for COVID-19 prevention and control	• 22% of the crew members of the USS Theodore Roosevelt were infected within five weeks<br>• Overall, two dozen crew members were hospitalized and one died<br>• 25 percent of the crew of the USS Kidd were infected<br>• Two examples of crowded living conditions, with limited access to health care, challenges regarding the personal situation on board, and an environment where it is impossible to maintain social distancing

Author, Year of Publication, Reference	Study Design; Method	Setting	Sample Size (*n*)	Population (crew /passengers)	Main Measurements	Aim of the Study	Main Results
Payne et al., 2020 [70]	Morbidity and Mortality Weekly Report; Questionnaires	Navy Aircraft Carrier (USS Theodore Roosevelt)	382 young adults survey participants, 238 (62.0%) participants with previous or current SARS-CoV-2 infection	Service members	PCR-Tests, antibody reactivity, neutralizing antibodies, demographic characteristics, exposure, COVID-19 protective behaviors, health history, symptoms, previous COVID-19 test since deployment	• To describe an outbreak of COVID-19 occurred during deployment	• 59 had reactive antibodies, and 59% of those also had neutralizing antibodies<br>• 20% were asymptomatic and two crew members (0.8%) were hospitalized<br>• Face coverings and social distancing had a positive impact for infection prevention
Plucinski et al., 2020 [44]	Research article; retrospective cross-sectional survey	Cruise ship (Diamond Princess)	114 confirmed infections of 437 tested Americans on the Diamond Princess	Passengers	Demographics, symptoms, comorbidities, smoking, participation in group events, the period just before ship-based quarantine, shore excursions during the voyage, close contacts during the ship-based	• To report about risk factors of infection and to describe transmission activities	• Attack rate in single-person cabins or without infected cabin mates was 18%, in contrast to 63% for those sharing a cabin with an asymptomatic infected cabinmates, and 81% for symptomatic infected cabinmates<br>• 14 (21%) were asymptomatic while on the ship (Of 66

Author, Year of Publication, Reference	Study Design; Method	Setting	Sample Size (*n*)	Population (crew /passengers)	Main Measurements	Aim of the Study	Main Results
					quarantine, PCR-Tests, cabin number, and symptom information at time of specimen collection		SARS-CoV-2-positive passengers)
Röcklöv et al., 2020 [32]	Research article; Statistical modelling, SEIR model to estimate basic reproduction number from the initial period of the outbreak	Cruise ship (Diamond Princess)	619 confirmed infection of SARS-CoV-2 of 3,700 passengers and crew members (as of 20 February 2020)	Passengers and crew members	Basic reproduction number	• To study empirical data of confirmed infections with SARS-CoV-2 on the Diamond Princess to estimate the basic reproduction number on cruise ships, the response effectiveness of the quarantine and removal interventions, and compare scenarios of an earlier and later evacuation	• 2,920 of 3,700 would have been infected by 19 February 2020 without any interventions<br>• Isolation and quarantine prevented 2,307 cases<br>• 76 infections with SARS-CoV-2 would have been estimated by means of an early evacuation of all passengers
Salducci et al., 2020 [53]	Research article; Case report	Cruise ship (Diamond Princess)	1 case with conjunctivitis	Passenger	Clinical course	• To examine the course of a COVID-19 case with a conjunctivitis	• Symptoms: swollen, red eyes, transparent serous secretions, conjunctival

Author, Year of Publication, Reference	Study Design; Method	Setting	Sample Size (*n*)	Population (crew /passengers)	Main Measurements	Aim of the Study	Main Results
							chemosis, pseudomembranes of fibrin and inflammatory cells on the tarsal conjunctiva accompanied by preauricular lymph nodes and enlarged submaxillaries
Schuchat A., 2020 [68]	Morbidity and Mortality Weekly Report	Cruise ship (Nile river cruise)	101 persons who had been on nine separate Nile River cruises	Passengers	Spread of Pandemic COVID-19 in the United States, from 24 February – 21 April 2020	• To highlight the risk of transmission by passengers returning from cruises<br>• To examine factors contributing to transmission and public health measures	• 101 persons were on nine different Nile River cruises during 11 February – 5 March and afterwards returned to 18 states
Sekizuka et al., 2020 [67]	Research article; Case report, Genome analysis	Cruise ship (Nile river cruise)	10 passengers	Passengers	Chapter Whole Genome Sequencing of SARS-CoV-2	• To examine SARS-CoV-2 genome of isolates from 10 travelers who returned from Egypt and from patients possibly associated with these passengers	• Results indicate two potential Egypt-related clusters which were related to globally detected viruses in different countries at that time of the pandemic

Author, Year of Publication, Reference	Study Design; Method	Setting	Sample Size (*n*)	Population (crew /passengers)	Main Measurements	Aim of the Study	Main Results
Sekizuka et al., 2020 [55]	Research article; Genome sequencing	Cruise ship (Diamond Princess)	Of 896 persons, PCR tests for SARS-CoV-2 were positive for 148 individuals (16.4%)	Passengers and crew members	Genome sequencing	• To highlight the usefulness of haplotype network/ phylogeny analysis for reconstructing infection routes	• The dissemination of SARS-CoV-2 resulted from a single introduction event before the quarantine started<br>• Some clusters were associated with transmission through mass-gathering events or direct transmission among passengers who shared cabins
Tabata et al., 2020 [45]	Research article; retrospective, single-center study	Cruise ship (Diamond Princess)	104, 43, 41, and 20 patients 40 on admission and 33, 43, and 28 patients at the end of observation had asymptomatic, mild, and 41 severe courses	Passengers	Clinical records, laboratory data, and radiological findings	• To examine the clinical characteristics on admission (of asymptomatic, mild, and severe COVID-19 cases) and at the end of observation	• LDH levels were found to be significantly higher in 10 patients who were asymptomatic on admission but developed symptoms compared with 33 patients who remained asymptomatic<br>• Risk factors: older age, consolidation on chest CT images, and lymphopenia
Yamagishi et al., 2020 [46]	Research article; Descriptive statistics	Cruise ship (Diamond Princess)	172 confirmed infections of	Crew members and passengers	PCR-Tests; general	• To inform about preliminary findings from the	• Attack rates were observed to be similar across

Author, Year of Publication, Reference	Study Design; Method	Setting	Sample Size (*n*)	Population (crew /passengers)	Main Measurements	Aim of the Study	Main Results
			490 tested for SARS-CoV-2		information (sex, age, nationality, screening data), Presence on ship, clinical symptoms, contact information from multiple sources	investigation on the Diamond Princess	the decks (most affected beverage (3.3%) and food service staff (5.7%)) and increased with age<br>• Outbreak response included surveillance, the provision of medical care, food and medicine delivery, isolation, infection prevention and control, sampling and disembarkation
Yamahata & Shibata, 2020 [47]	Viewpoint; medical activities on the Diamond Princess	Cruise ship (Diamond Princess)	PCR-tests for SARS-CoV-2 were conducted for 3,711 persons on board (2,666 passengers and 1,045 crew), 696 were tested positive	Crew members and passengers	PCR-Tests	• To report about the medical activities and difficulties in implementing quarantine on a cruise ship	• 410 were asymptomatic and 54% of the asymptomatic patients had lung opacities on chest CT<br>• Difficulties in implementing quarantine: creating traffic line between infectious and noninfectious passengers, identifying hospital and transportation

Author, Year of Publication, Reference	Study Design; Method	Setting	Sample Size (*n*)	Population (crew /passengers)	Main Measurements	Aim of the Study	Main Results
							providers, language barriers, and maintenance of the daily life
Yoshimura et al., 2020 [52]	Research article; Single-center, retrospective, observational study	Cruise ship (Diamond Princess)	17 COVID-19 cases, symptomatic patients who were infected on the ship and admitted to the hospital between 5 and 19 February 2020	Passengers	PCR-test results, Sex, age, race, residence, comorbidities, Charlson Comorbidity Index, period from symptom onset to admission, Symptoms, Chest X-ray, Chest computed tomography, Chest-x-ray	• To describe the clinical characteristics of the COVID-19 cases	• Major symptoms: cough and fever<br>• CT scans shows bilateral GGO predominantly in the peripheral area<br>• CRP levels higher in severe and critical cases compared to mild to moderate cases
Zhang et al., 2020 [62]	Research article; Mathematical modelling, serial interval (mean and standard deviation) with a gamma distribution	Cruise ship (Diamond Princess)	355 confirmed infections with SARS-CoV-2 identified on the ship on 16 February 2020	Crew members and passengers	Serial interval, with Maximum-Likelihood (ML) estimation	• To estimate the reproductive number (R0) during an early stage of outbreak and make a prediction of daily new cases on the ship	• Maximum-Likelihood (ML) value of R0 was 2.28 at the early stage on the ship<br>• Probable number of new cases in ten days would gradually increase, wherefore the estimated cumulative cases would be 1514 at the tenth day

Author, Year of Publication, Reference	Study Design; Method	Setting	Sample Size (*n*)	Population (crew /passengers)	Main Measurements	Aim of the Study	Main Results
							• Estimated cumulative cases would be reduced to 1,081 (981–1,177) and 758 (697–817), respectively, if R0 value was reduced by 25% and 50%

Abbreviations: AST: aspartate aminotransferase; COVID-19: Coronavirus Disease 2019; CrI: credible interval; CRP: C-reactive protein; CT: computer tomography; DMAT: Japanese Disaster Medical Assistance Team; GGO: Ground-glass opacity; IgG: Immunoglobulin G; IgM: Immunoglobulin M; LDH: Serum lactate hydrogenase; ML: Maximum-Likelihood; NT: Northern Territory; OD: optical density; PaO_2: Arterial partial pressure of oxygen; PCR: polymerase chain reaction; R0: reproductive number; RBD: receptor-binding protein; SARS-CoV-2: RNA: ribonucleic acid; Severe acute respiratory syndrome coronavirus type 2; SPO_2: Oxygen saturation
